# Integrated physiological, microbial, and metabolomics analyses revealed the differences in different varieties of *Paeonia lactiflora* Pall

**DOI:** 10.3389/fpls.2025.1577695

**Published:** 2025-05-23

**Authors:** Shuang Wu, Beijing Tian, Chenggang Shan, Xin Wang, Xinjing Xie, Hongqing Xie, Xiuwen Jia, Feng Zhang, Jinlong Han

**Affiliations:** ^1^ Institute of Industry Crops, Shandong Academy of Agricultural Sciences, Jinan, Shandong, China; ^2^ National Center of Technology Innovation for Comprehensive Utilization of Saline-Alkali Land, Dongying, China; ^3^ Shandong Xieshi Chinese Herbal Pieces Co., Ltd., Heze, China

**Keywords:** *Paeonia lactiflora* Pall., metabolome, microbiome, active ingredient, antioxidant activity

## Abstract

*Paeonia lactiflora* Pall (*P. lactiflora*) is a perennial herb with high medicinal and economic value. In the growth process of *P. lactiflora*, the plant’s root secondary metabolism is intricately linked to the microbial communities that surround it. However, few systematic studies have reported the changes in the microbiome and metabolites during *P. lactiflora* cultivation thus far. In this study, amplicon sequencing technology was used to determine the difference in rhizosphere microorganisms of *P. lactiflora*. The non-targeted metabolomics method was used to determine the changes in root metabolites, and the relationship between microorganisms and metabolites was demonstrated by co-expression network analysis. The paeoniflorin content (PC) was determined by HPLC. The total phenol content (TPC) was determined by the Folin–Ciocalteu method, and the total flavonoid content (TFC) was determined by the NaNO_2_-Al (NO_3_)_3_ method. The antioxidants were evaluated with the DPPH, ABTS, and FRAP methods. Results showed that Proteobacteria had the highest relative abundance among all phyla, Halomonas had the highest relative abundance among all genera. The results of metabolomics showed that 693 metabolites and 207 differential metabolites were detected in the four groups, which were mainly enriched in the biosynthesis of phenylpropanoids, phenylpropanoid biosynthesis, taste transduction, central carbon metabolism in cancer, and biosynthesis of plant secondary metabolites. The results also showed that the PC, TPC, TFC, and antioxidant capacity of the white *P. lactiflora* group were higher than those of the other groups. This study revealed the differences between different varieties of *P. lactiflora* and provided theoretical support for breeding and data reference for improving the quality of *P. lactiflora* by regulating microbial species.

## 1 Introduction

Medicinal plants are invaluable sources of bioactive compounds, whose therapeutic potentials are largely determined by their secondary metabolites. As one of the most iconic ornamental and medicinal plants*, P. lactiflora* belonging to the *Paeonia* genus is a traditional Chinese medicine in China, and it has high economic and medical value (Liang et al., 2023). It is naturally widely distributed in Shandong, Anhui, Zhejiang, and other provinces (Dong et al., 2023). As a valuable economic crop, all parts are high-quality medicine materials with valuable development potential and have been used for over 1000 years (Xiaowen et al., 2022). Numerous botanical studies have shown that monoterpenes, polyphenols, lipids, flavonoids, and steroids are the main biologically active substances of *P. lactiflora* (Li et al., 2021; Xu et al., 2024). According to research, the *P. lactiflora* extract has a wide range of biological activities, including neuroprotection (Sun et al., 2023), anti-inflammatory (Chen et al., 2024b), renal (Xia et al., 2022), and hypoglycemic functions (Yang et al., 2022). In addition, it is known to protect against pancreatic cancer (Cao et al., 2022) and treat stomach cancer (Zhou et al., 2023).

Although the metabolites of medicinal plants are abundant, they are greatly affected by soil microorganisms (Kashyap et al., 2022). There is a close relationship between rhizosphere microorganisms and plant growth (Ganugi et al., 2023). Rhizosphere microorganisms can promote plant growth by forming a symbiotic relationship with plant roots. For example, rhizosphere microorganisms can enhance the stress resistance of plants (Chen et al., 2024a), improve the absorption of nutrients by plants (Sarkar et al., 2022), and enhance the resistance of medicinal plants to pests and diseases (Zhang et al., 2021a). Studies have also found that rhizosphere microorganisms can affect the metabolites of plants. For example, rhizosphere microorganisms affect seven active components in *Acanthopanax* (Yuan et al., 2024), atractylone in *Atractylodes* macrocephalae (Song et al., 2023), and atractylodin and atractylon in *Atractylodes* (Tang et al., 2023).

Although previous studies have characterized the bioactive constituents of *P. lactiflora*, most research has focused on single compounds or limited varieties, neglecting systematic comparisons across different varieties (Čutović et al., 2022; Zhao et al., 2023). Little is known about how flower color correlates with comprehensive metabolite profiles, rhizosphere microbial communities, and their combined effects on medicinal quality. In particular, the potential interactions between soil microbiota and host metabolic biosynthesis—a critical factor influencing plant chemotypes—remain unexplored in *P. lactiflora*. It is widely known that the microbial species, metabolites, and antioxidant activities of medicinal plants from different sources vary. By contrast, the types of microorganisms and metabolites also affect the quality of medicinal materials. Studies have shown that different varieties of *Eucommia ulmoides* Oliv. metabolites are distinct, especially flavonoid metabolites (Du et al., 2022). Tryptophan and terpenoids of different varieties of *Cinnamon* are significantly different (Zhao et al., 2022), and rhizosphere microorganisms of varying blueberry varieties also differ (Zhang et al., 2021b). Antioxidant activity refers to the ability of substances to resist the harmful effects of free radicals or other oxidizing substances. In organisms, free radicals produced by oxidation reactions can damage cell structure and function, leading to cell senescence and disease. Therefore, substances with good antioxidant activity can help protect cells from oxidative damage and maintain cell health and normal function (Nwozo et al., 2023). The antioxidant activities of different varieties of hemp leaf extracts vary (Stasiłowicz-Krzemień et al., 2023). For lotus seeds, the antioxidant activities were evaluated by DPPH, FRAP, and ABTS assays, with the cultivar Taikong 66 showing the highest antioxidant activities among the tested cultivars (Yu et al., 2024).

In this study, 16S RNA and non-target metabolomics methods were used to detect the changes in rhizosphere microorganisms and root metabolites of 4-year-old *P. lactiflora* with different varieties, and the interaction between microorganisms and metabolites was analyzed. The paeoniflorin content (PC), total phenolic content (TPC), total flavonoid content (TFC), DPPH, FRAP, and ABTS assays were compared. This study explored the differences between peonies with different varieties and provided data support for the breeding of *P. lactiflora*.

## 2 Materials and methods

### 2.1 Sample collection

**Figure 1:**
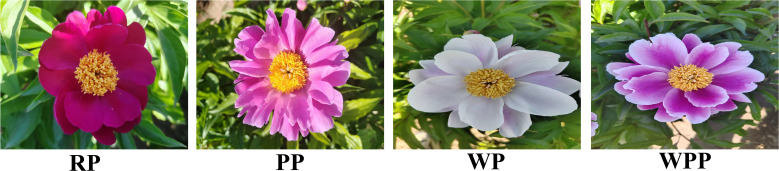
Different varieties of *P. lactiflora*. RP, red *P. lactiflora*; PP, pink *P. lactiflora*; WP, white *P. lactiflora*; WPP, white and pink *P. lactiflora*.

The samples were collected from Liuzhuang Village, Penglou Town, Juancheng County, Heze City, Shandong Province, China (35°25′8.74″N, 115°34′44.9″E). We selected 4-year-old medicinal peonies with different varieties in May 2023 (**Figure 1**, RP-red *P. lactiflora*, herbarium number CS2021036; PP- pink *P. lactiflora*, herbarium number CS2021037; WP-white *P. lactiflora*, herbarium number CS2021038; WPP-white and pink *P. lactiflora*, herbarium number CS2021038). All four *P. lactiflora* varieties with distinct varieties were derived from natural variations of wild *P. lactiflora* through artificial domestication. These varieties have been cultivated locally for over 20 years, demonstrating stable phenotypic characteristics throughout this extended period. The fresh plants were uprooted, the loose soil on the root surface was removed, and the inter-root soil, which was closely associated with the root, was collected with a sterile brush and stored in a −80°C refrigerator for microbial analysis. The root samples of *P. lactiflora* were cleaned and stored in an ultra-low temperature refrigerator at -80°C for metabolite analysis. The root of *P. lactiflora* collected by the sample was washed with distilled water, dried, crushed, and reserved for PC, TPC, TFC and antioxidant activity determination. Each treatment consisted of three replicates.

### 2.2 Soil microbe extraction and sequencing

The fecal genome DNA extraction kit (DP328) was used to extract DNA from samples. Amplicon library and sequencing amplicon libraries were constructed with Illumina sequencing-compatible and barcode-indexed bacterial PCR primers B341F/B785R, which target the V3–V4 regions of the 16S rRNA gene (20). All PCR reactions were performed with 2×phanta max master mix using the manufacturer’s protocol (Vazyme Biotech Co., Ltd.) and approximately 50 ng of extracted DNA per reaction. The thermocycling conditions were set at 95°C for 1 min, 55°C for 1 min, 72°C for 1 min for 30 cycles, and a final extension at 72°C for 5 min. All PCR reactions were performed in 50 mL triplicate and combined after PCR. The amplicon library was prepared using a TruSeqDNA sample preparation kit (Illumina Inc., San Diego, CA, USA). Before sequencing, the PCR products were extracted with the MiniElute Gel Extraction Kit (QIAGEN) and quantified on a NanoDrop ND-1000 spectrophotometer (Thermo Electron Corporation) and Qubit 2.0 Fluorometer (Invitrogen). The purified amplicons were then pooled in equimolar concentrations, and the final concentration of the library was determined by Qubit (Invitrogen). Negative DNA extraction controls (lysis buffer and kit reagents only) were amplified and sequenced as contamination controls. Sequencing was performed on a Novaseq instrument (Illumina) using a PE250 kit.

### 2.3 Metabolome extraction and sequencing

#### 2.3.1 Metabolite extraction

We accurately weighed an appropriate amount of sample into a 2 mL centrifuge tube, which was added with 600 µL of MeOH (containing 2-Amino-3-(2-chloro-phenyl)-propionic acid (4 ppm). The tube was vortexed for 30 s, added with steel balls, placed in a tissue grinder for 60 s at 55 Hz, subjected to room-temperature ultrasound for 15 min, and centrifuged for 10 min at 12,000 rpm and 4 °C. The supernatant was filtered by using a 0.22 μm membrane and transferred into a detection bottle for LC–MS detection.

#### 2.3.2 Liquid chromatography conditions

The LC analysis was conducted using a Vanquish UHPLC system (Thermo Fisher Scientific, USA) equipped with an ACQUITY UPLC^®^ HSS T3 column (2.1 × 100 mm, 1.8 µm; Waters, Milford, MA, USA). The column temperature was maintained at 40°C, with a flow rate of 0.3 mL/min and an injection volume of 2 μL. For LC-ESI (+)-MS analysis, the mobile phases consisted of (A2) 0.1% formic acid in water (v/v) and (B2) 0.1% formic acid in acetonitrile (v/v), with the following gradient program: 0–1 min, 8% B2; 1–8 min, 8%–98% B2; 8–10 min, 98% B2; 10–10.1 min, 98%–8% B2; and 10.1–12 min, 8% B2. For LC-ESI (-)-MS analysis, the mobile phases were (A3) 5 mM ammonium formate and (B3) acetonitrile, using the same gradient profile: 0–1 min, 8% B3; 1–8 min, 8%–98% B3; 8–10 min, 98% B3; 10–10.1 min, 98%–8% B3; and 10.1–12 min, 8% B3.

#### 2.3.3 Mass spectrum conditions

Mass spectrometric analysis was conducted using an Orbitrap Exploris 120 mass spectrometer (Thermo Fisher Scientific, USA) equipped with an electrospray ionization (ESI) source. Data acquisition was performed in Full MS-ddMS² mode, enabling simultaneous MS¹ and data-dependent MS/MS scans. The instrument parameters were optimized as follows: sheath gas pressure, 40 arb; auxiliary gas flow, 10 arb; spray voltage, 3.50 kV (positive mode) and -2.50 kV (negative mode); capillary temperature, 325 C; MS¹ scan range, m/z 100–1000 at a resolution of 60,000 FWHM; data-dependent acquisition of top 4 most intense ions per cycle with MS/MS scans at 15,000 FWHM resolution; normalized collision energy, 30%; and automatic dynamic exclusion.

### 2.4 Active ingredient determination

The PC determination: According to the Chinese Pharmacopoeia 2020 method (Pharmacopoeia of the People’s Republic of China (I), 2020), 0.1 g of the powder was accurately measured and placed in a 50 mL volumetric flask. About 35 mL of dilute ethanol was added. The mixture was subjected to ultrasonic treatment (240 W and 45 kHz) for 30 min. After cooling, the solution was further diluted with dilute ethanol to reach the desired volume on the flask’s scale. The mixture was shaken and filtered, and the filtrate was obtained. The mobile phase was acetonitrile-0.1–0.1% phosphoric acid solution (14:86), the detection wavelength was 230, the flow rate was 1.0 mL/min, and the column temperature was 25°C.

TPC determination: The TPC was determined using an improved Folin–Ciocalteu method (Cai et al., 2024). Specifically, 0.4 mL of the sample solution was diluted with 0.6 mL of distilled water, followed by the addition of 1 mL of Folin–Ciocalteu reagent (10% concentration). After thorough mixing for 5 min, 1 mL of Na_2_CO_3_ solution (15% concentration) was added and the mixture was mixed again. The mixture was incubated at 30°C for 60 min. Subsequently, the absorbance of the solution was measured at 765 nm using a spectrophotometer, with gallic acid used as the standard. The regression equation of the standard curve was Y=10.725x+0.0049 (R^2^ = 0.9994). This chemical analysis method allows for the precise determination of TPC in different samples.

TFC determination: TFC was determined using an improved NaNO_2_-Al (NO_3_)_3_ method (Keivani et al., 2024). The specific operational steps were as follows: 0.1 mL of the sample solution was diluted with 0.9 mL of distilled water, followed by the addition of 0.5 mL of NaNO_2_ (5%). After thorough mixing for 6 min, 0.5 mL of Al (NO_3_)_3_ solution (1%) was added, and the reaction was continued for 6 min. After thorough mixing again, 1 mL of NaOH (5%) was added, and the reaction was continued for 15 min. Subsequently, the absorbance of the solution was measured at 510 nm using a spectrophotometer, with rutin used as the standard. The regression equation of the standard curve was Y=0.7125x−0.0004 (R^2^ = 0.9994). This chemical analysis method allows for the precise determination of TFC in different samples.

### 2.5 Antioxidant activity determination

Determination of DPPH free radical scavenging capacity (Grzeszczak et al., 2024): 1 mg of DPPH was weighed in a brown bottle and dissolved in 30 mL of ethanol. The 0.5 mL sample was added to a 5 mL centrifuge tube, and 1 mL of DPPH was added. The volume of anhydrous ethanol was fixed at 3 mL. After fully mixing, the reaction was kept away from light for 70 min. The absorbance was measured at 517 nm. The absorbance of DPPH and anhydrous ethanol was the initial absorbance value.


$$Y=\frac{1-A_{1}}{A_{0}}\times 100\%$$

Y— DPPH free radical scavenging capacity; A_1_—Absorbance of the sample solution; A_0_—Initial absorbance value.

Determination of ABTS free radical scavenging ability (Del Sole et al., 2024): According to the requirements of the kit, 100 μL of ABTS and ABTS oxidant solutions were placed in the dark at room temperature for 18 h, and 80% ethanol was used to dilute the ABTS mother liquor to obtain the ABTS working solution. About 200 μL of ABTS working solution and 10 μL of sample solution were mixed in 96-well plates and left to stand in the dark for 6 min at room temperature, and the absorbance was measured at 734 nm. ABTS working solution and sample solution were substituted with dilute ethanol.


$$Y=1-\frac{A_{1}-A_{2}}{A_{0}}\times 100\%$$

Y—ABTS free radical scavenging ability; A_0_—Absorbance of methanol instead of sample solution; A_1_—Absorbance of the sample solution; A_2_—Absorbance of methanol instead of ABTS working solution.

Total antioxidant capacity (Rumpf et al., 2023): According to the kit requirements, the concentration of Fe·7H_2_O dissolved in anhydrous ethanol was 100 mmol/L. Subsequently, 2,4,6-tripyridyl-s-triazine (15 mL), TPTZ (15 mL), and detection buffer (1.5 mL) were mixed to make a FRAP working solution. About 180 μL of FRAP working solution and 5 μL of sample solution, and ethanol solution were added to the 96-well plate, and the ethanol solution was used as the negative control. After mixing, the plate was placed at 37°C for 5 min, and the absorbance was measured at 593 nm.

### 2.6 Statistical analysis

Metabolomics analysis of samples detected by LC–MS/MS was performed. The process involved sequencing a highly variable region of 16S rRNA, which encodes the small subunit rRNA in eukaryotes. All experiments were performed in triplicate. Data are shown as mean ± standard deviation (SD). The mean and SD were calculated by multiple comparison analysis and ANOVA for statistical tests. Graphs were drawn using OriginPro (version 2021).

we converted the raw mass spectrometry data (e.g., raw or.wiff formats) to open. mzML/.mzXML formats using ProteoWizard MSConvert for subsequent analysis. Next, we performed data preprocessing, including peak picking, alignment, and normalization using either XCMS Online or MS-DIAL. Metabolite identification was conducted by matching characteristic peaks and MS/MS fragmentation patterns against major public databases, including HMDB (http://www.hmdb.ca/) and METLIN (https://metlin.scripps.edu/). Finally, we performed multivariate statistical analysis (PCA, PLS-DA) and hypothesis testing (t-tests, volcano plots) using R packages (MetaboAnalystR or limma), and generated visualizations such as heatmaps and boxplots using OriginPro (version 2021). The total ion current diagram is shown in **Supplementary Figures 1**-**5**.

## 3 Results

### 3.1 Effects of varieties on the soil microbes of *P. lactiflora*

#### 3.1.1 Taxonomic composition analysis

**Figure 2:**
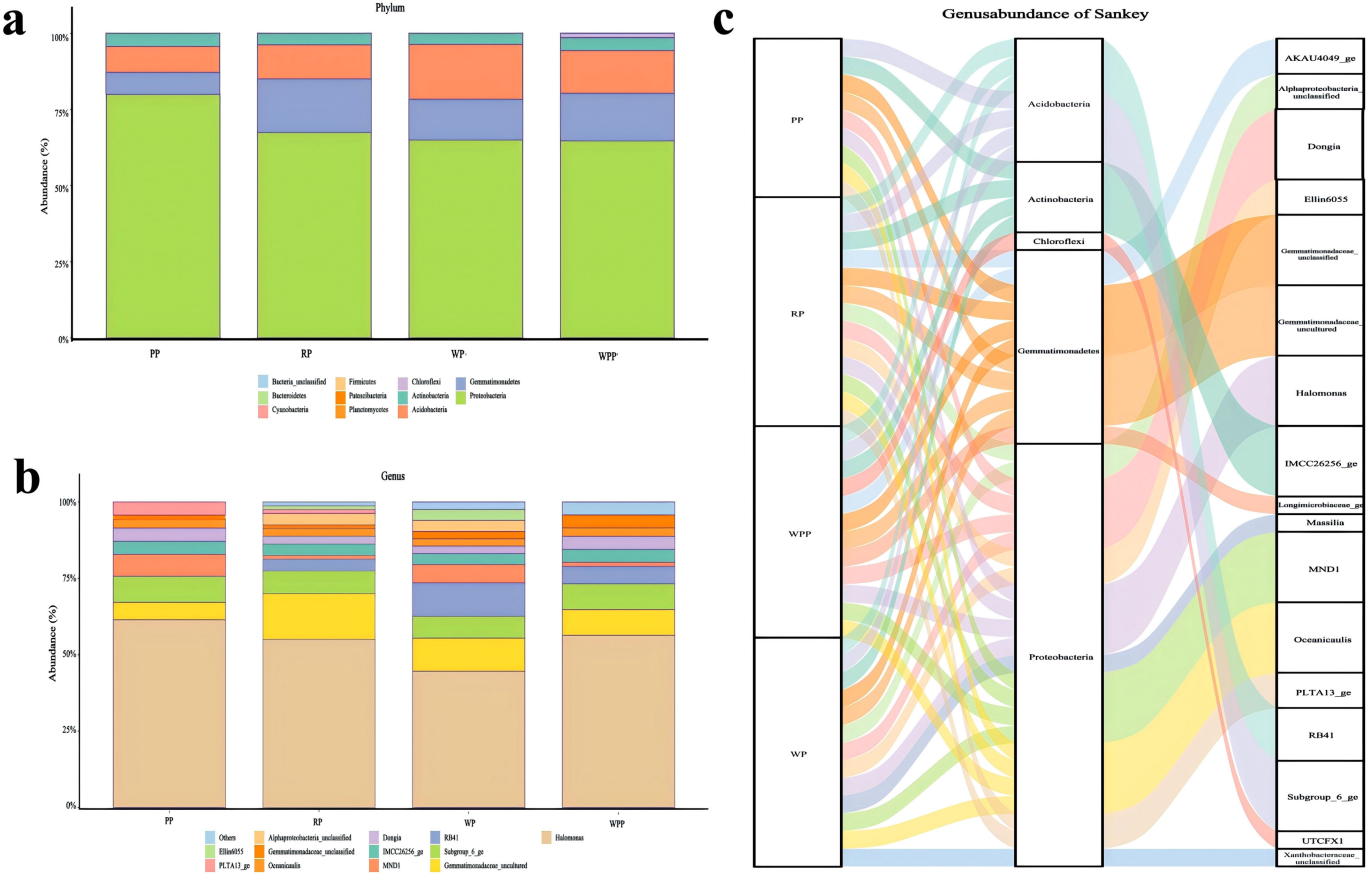
Inter-root microbial communities. **(a)** Relative abundance of bacteria at the phylum level. **(b)** Relative abundance of bacteria at the genus level. **(c)** Combining bacterial abundance at the phylum and genus levels.

Bulleted lists look like this: After Illumina sequencing, the results of sequencing 12 samples showed that a total of 741,216 raw reads were obtained. To obtain high-quality sequencing data to improve the accuracy of subsequent bioinformatic analyses, we first spliced the raw downlinked data and then carried out quality control and filtration to obtain 454,633 clean reads (**Supplementary Table 1**). We analyzed the composition of rhizosphere microorganisms at the phylum and genus levels. A total of 4, 4, 4, and 5 (PP, RP, WP, and WPP, respectively) taxa (**Supplementary Table 2**) were detected at the phylum level, and 9, 13, 13, and 12 (PP, RP, WP, and WPP, respectively) taxa (**Supplementary Table 3**) were detected at the genus level. As shown in **Figure 2a**, *Proteobacteria*, *Gemmatimonadetes*, *Acidobacteria*, *Actinobacteria*, and *Chloroflexi* at the phylum level (**Figure 2a**) represented about 99% of the microorganisms detected in 12 soil samples, and Proteobacteria had the highest relative abundance among all phyla. As shown in **Figure 2b**, Halomonas, *Subgroup_6_ge*, *Gemmatimonadaceae_uncultured*, *MND1*, *Gemmati-monadaceae_unclassified*, *IMCC26256_ge*, *Dongia*, and *Oceanicaulis* at the genus level represented about 95% of the microorganisms detected in 12 soil samples. *Halomonas* had the highest relative abundance among all genera (**Figure 2c**).

#### 3.1.2 Bacterial alpha-diversity and beta-diversity

**Figure 3:**
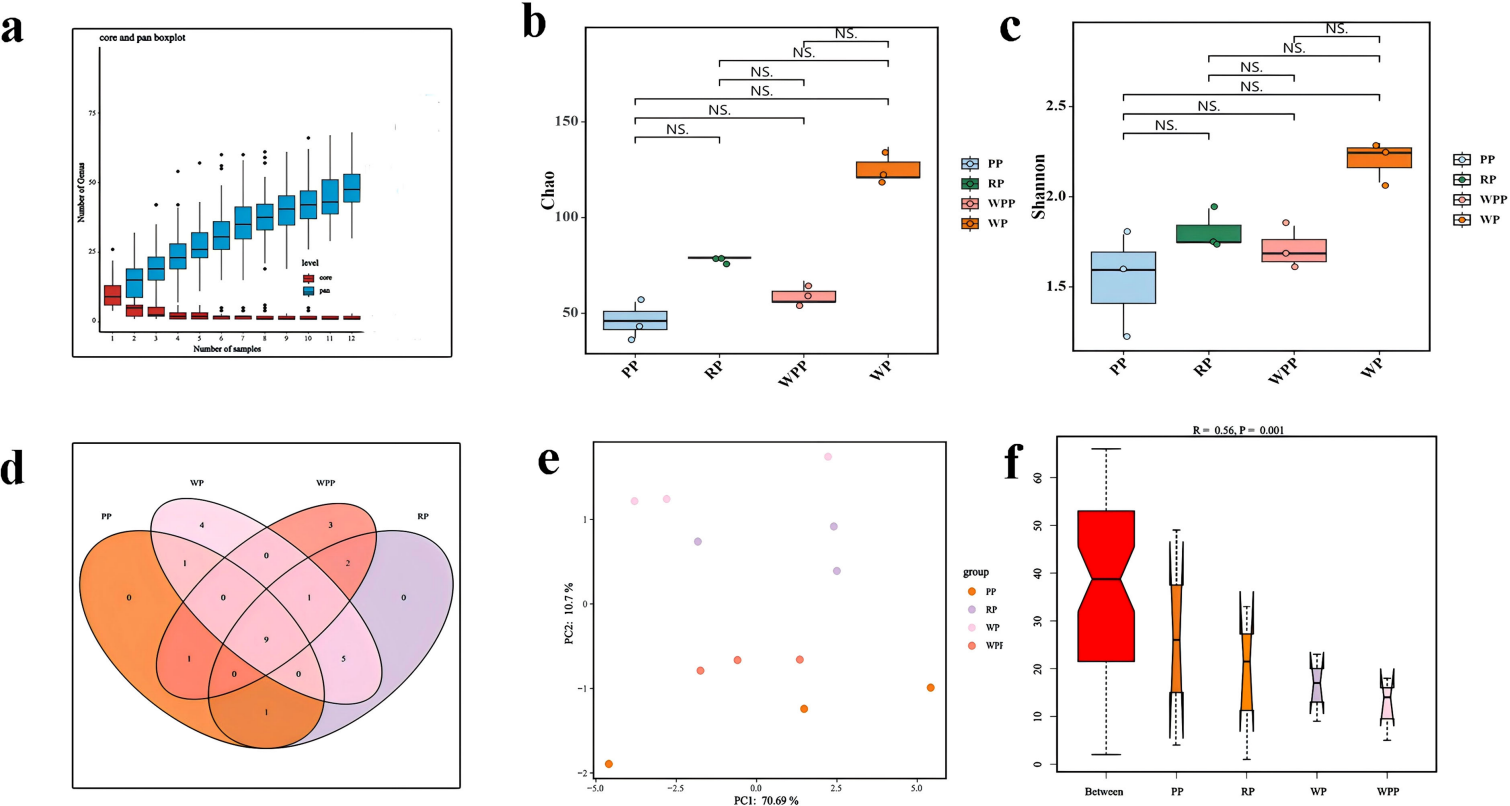
Bacterial alpha-diversity and beta-diversity. **(a)** Core- pan species analysis. **(b)** Chao index. **(c)** Shannon index. **(d)** Venn diagram of OTU. **(e)** PCA analysis of bacterial communities in four groups. **(f)** Analysis of similarities.

Pan/core species analyses are used to describe changes in total and core species amounts as the sample size increases. **Figure 3a** shows that our sample size was sufficient to assess total species richness and the number of core species in the environment. As shown in **Figure 3b**, the Chao and Shannon indices of bacterial flora among the PP, RP, WPP, and WP groups were not statistically significant, but the differences varied significantly. The Chao indices were 46, 79, 60, and 126 for the PP, RP, WPP, and WP groups, respectively, and the Shannon indices were 1.54, 1.81, 1.71, and 2.21 for the PP, RP, WPP, and WP groups, respectively (**Figure 3c**). These results indicated that the total number of inter-root species and community diversity index were highest in the WP group, whereas the total number of rhizosphere species and community diversity index were lowest in the PP group during the growth of *P. lactiflora*.

Classification by operational taxonomic unit (OTU) revealed that the PP group had 12 OTUs, the RP group had 18 OTUs, the WPP group had 16 OTUs, and the WP group had 20 OTUs. The four groups had a total of 9 groups of OTUs (**Figure 3d**). Principal component analysis (PCA) was utilized to analyze the PP, RP, WP, and WPP groups (**Figure 3e**). The WP group was located in the upper part of the graph, the PP group was located in the bottom part of the graph, and the RP and WPP groups were located in the middle part of the graph. The distribution of different samples was discrete. PC1 accounted for 70.69% of the total variation, whereas PC2 accounted for 10.7%. The results showed a distinctive difference in bacterial community structures. The similarity between groups of high-dimensional data was analyzed to provide a basis for the evaluation of the significance of differences between data. As shown in **Figure 3f**, the high rank of between-group similarity among every subgroup indicated that the bacterial communities exhibited many differences among the four groups (R=0.564). Thus, the intergroup differences among the four subgroups were significantly greater than the intragroup differences (p=0.0001).

**Figure 4:**
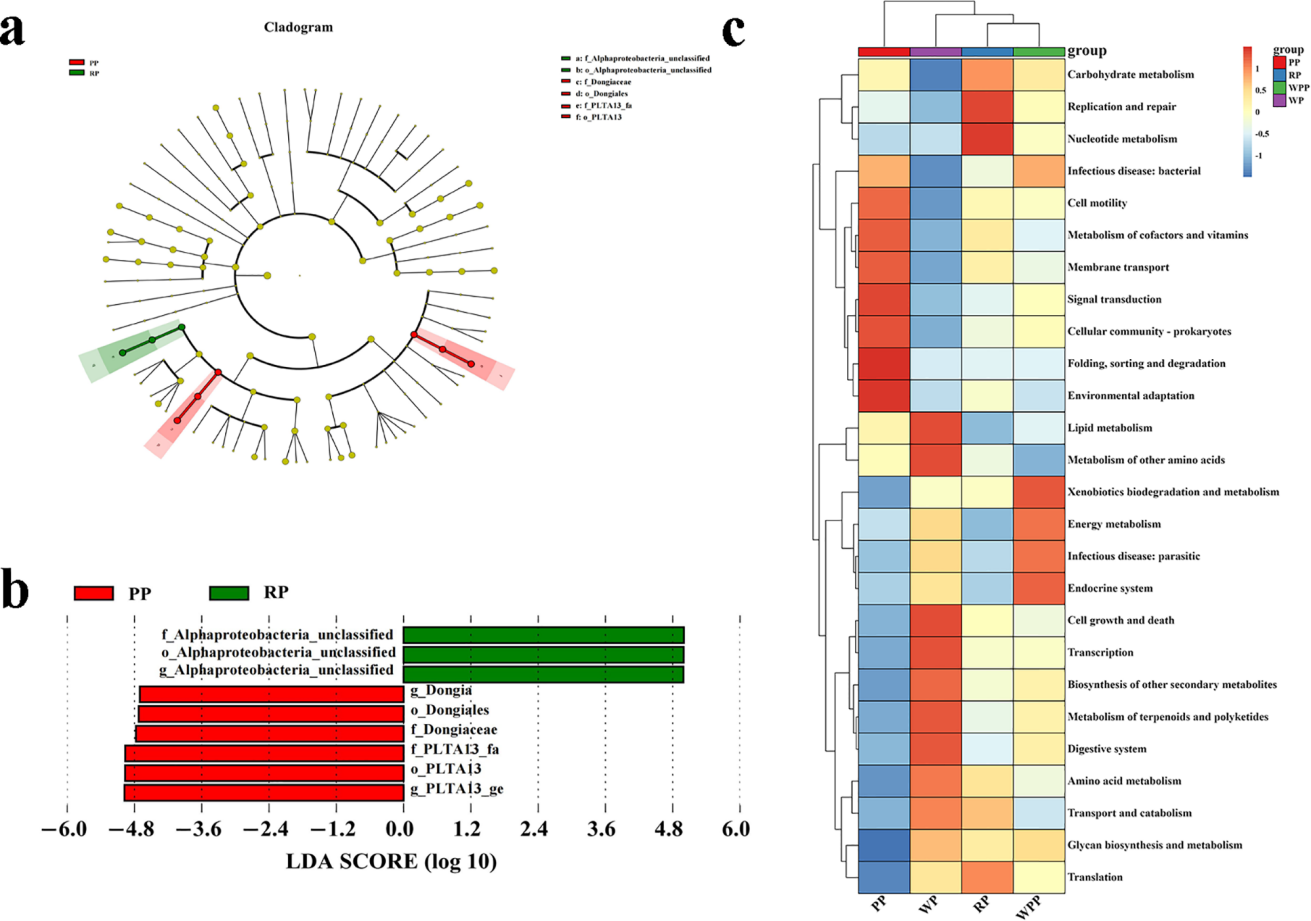
Differential and functional analyses of inter-root microorganisms. **(a)** LEfSe analysis showed changes in the abundance at different taxonomic levels. **(b)** Differential species with significantly high abundance. **(c)** Functional heatmap of a microbial community.

LEfSe analysis revealed that the relative abundance of bacterial species among the four groups significantly differed in only two groups (**Figures 4a, b**, **Supplementary Table 4**). Nine biomarkers were enriched in the samples with an LDA score >4 (**Supplementary Table 4**). Among these biomarkers, six taxa were present in PSL with major contributions: *g_PLTA13_ge*, *o_PLTA13*, *f_PLTA13_fa*, *f_Dongiaceae*, *o_Dongiales*, and *g_Dongia*. Three taxa were present in RP with major contributions: *f_Alphaproteobacteria _unclassified*, *o_Alphaproteobacteria _unclassified*, and *g_Alphaproteobacteria _unclassified*.

#### 3.1.3 Potential functional pathways in rhizosphere soil microbes

As illustrated by the Venn diagram (**Supplementary Figure 6**), 26 level 2 pathways were annotated in the KEGG database for all samples, of which 25, 25, 26, and 25 pathways were detected in PP, RP, WPP, and WP, respectively. Among them, there was 1 unique pathway in WPP. The annotation results showed that carbohydrate metabolism, biosynthesis of other secondary metabolites, metabolism of cofactors and vitamins, lipid metabolism, and energy metabolism represented 14.36%, 14.35%, 13.71%, 7.14%, and 5.23% of the total (**Supplementary Table 5**), respectively. Therefore, the data were plotted as a functional heatmap (**Figure 4c**). The results showed that the PP soil was enriched with the denitrification of folding, sorting, and degradation; environmental adaptation; and signal transduction. WP was enriched with lipid metabolism and the metabolism of other amino acids. RP was enriched with replication and repair, and nucleotide metabolism. WPP was enriched with xenobiotic biodegradation and metabolism, and the endocrine system.

### 3.2 Effects of varieties on the soil microbes of *P. lactiflora*

**Figure 5:**
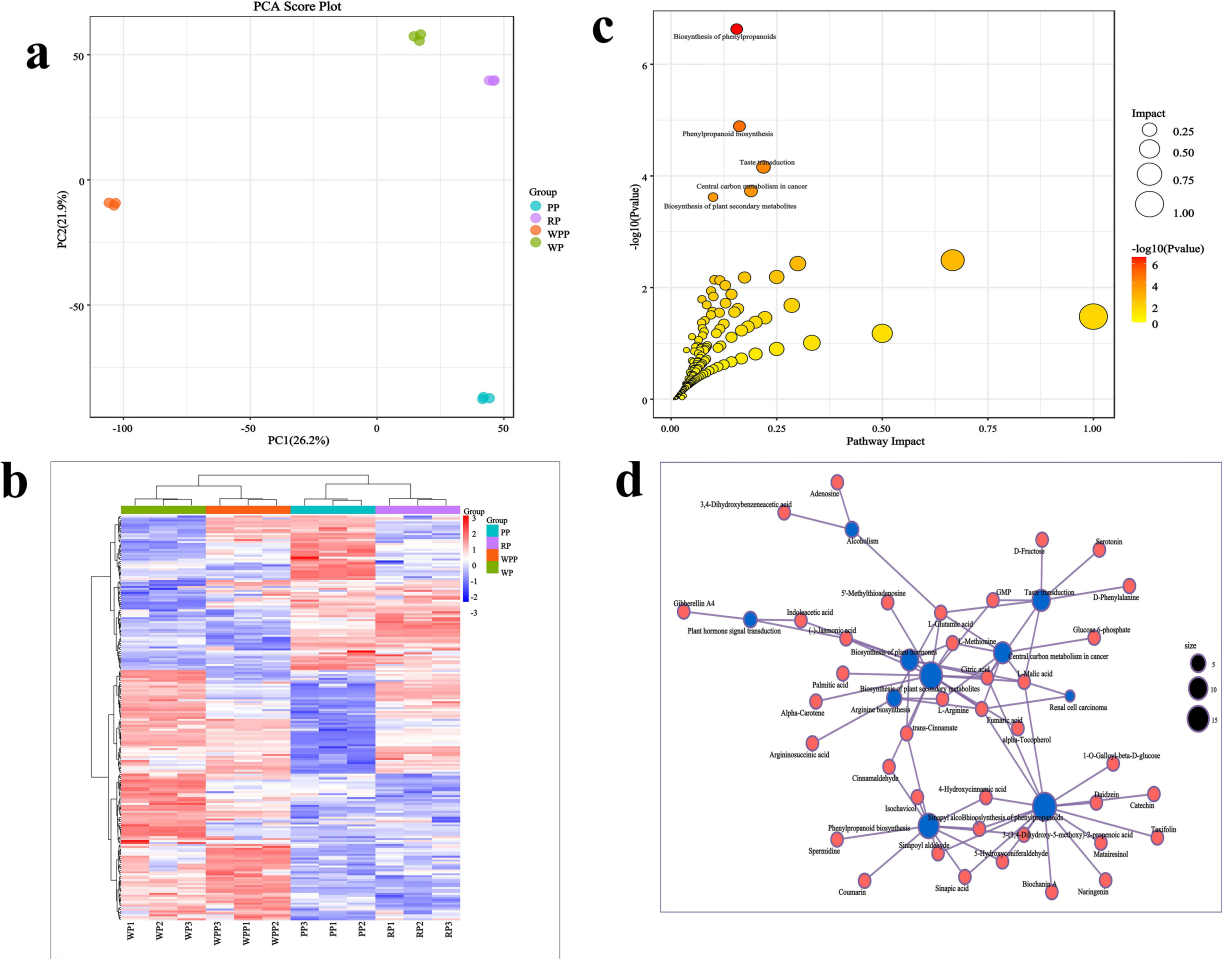
Changes in root metabolites in four varieties. **(a)** Principal component analysis. **(b)** Clustering analysis of differentially abundant metabolites. **(c)** Enrichment analysis. **(d)** Correlation analysis of differential metabolites and metabolic pathways.

The results of PCA for metabolites indicated that samples containing 12 metabolites were significantly categorized into four groups, with distinct differences observed among the metabolites in each group. PC1 and PC2 explained 26.2% and 21.9% of the total variance, respectively (**Figure 5a**). A total of 693 metabolites were identified across all samples (**Supplementary Table 5**), among which 207 were found to be significantly differentiated. Among these differentiated metabolites, acids were the most abundant (34 compounds), followed by ketones (23 compounds), amino acid derivatives (22 compounds), alcohols (18 compounds), and glycosides (15 compounds). Hierarchical cluster analysis of these 207 differentiated metabolites (**Figure 5b**) further categorized them into four distinct clusters. WP highly expressed an average of 42 metabolites, WPP expressed 41 metabolites, PP expressed 29 metabolites, and RP expressed 39 metabolites. These differentially expressed metabolites exhibited synergistic or mutually exclusive relationships, with positive correlations nearing 1 and negative correlations nearing −1, all of which were statistically significant (P < 0.05). Among the differential metabolites, 422 groups showed significant positive correlations, whereas 110 metabolites exhibited significant negative correlations (**Supplementary Table 6**). Metabolic pathway analysis (**Figure 5c**) revealed that 207 metabolites were predominantly involved in pathways such as biosynthesis of phenylpropanoids, phenylpropanoid biosynthesis, taste transduction, central carbon metabolism in cancer, and bio-synthesis of plant secondary metabolites. Notably, citric acid, L-malic acid, (-)-jasmonic acid, L-methionine, fumaric acid, and trans-cinnamate were found in multiple pathways (**Figure 5d**).

### 3.3 Correlations between soil microbes and metabolites

**Figure 6:**
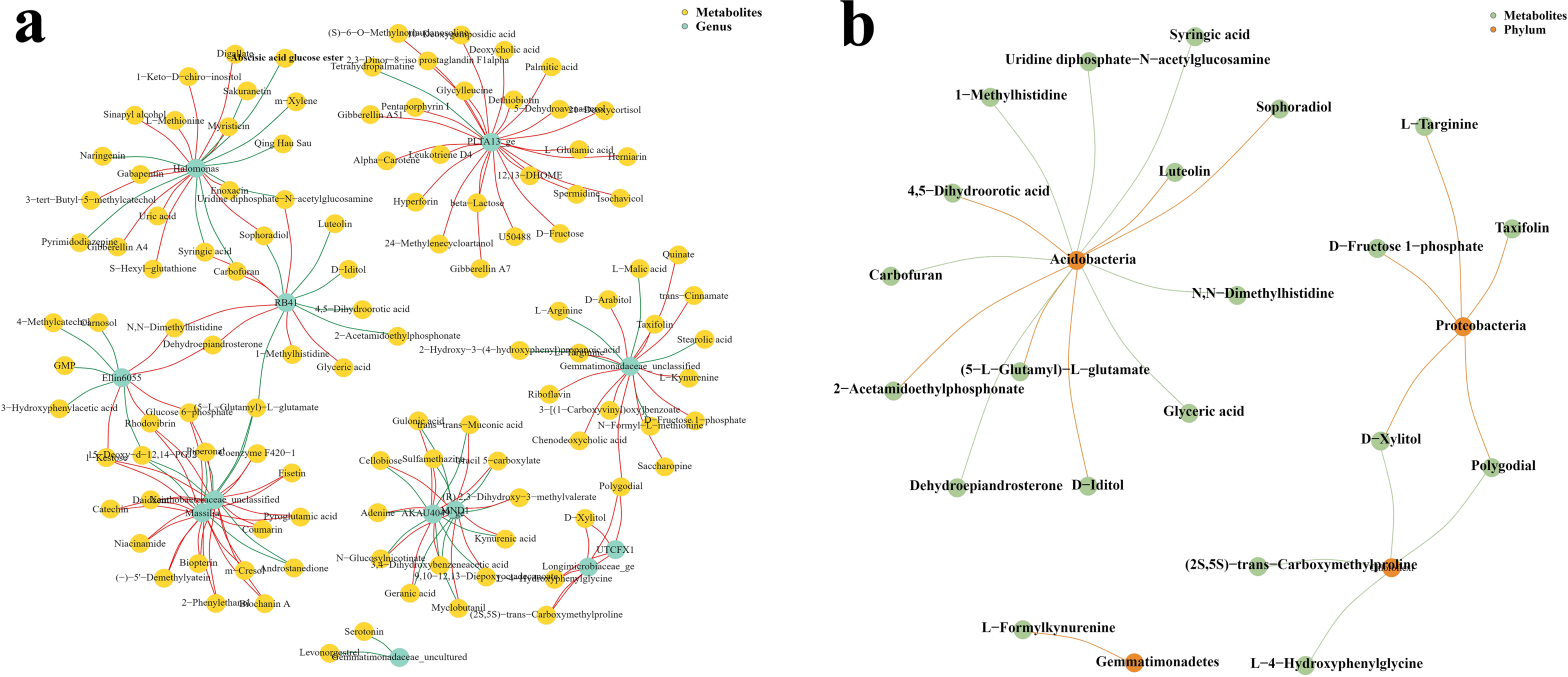
Co-occurrence network maps of differential microbial communities and metabolites at the genus and phylum levels. **(a)** Genus level. **(b)** Phylum levels.

At the genus level (**Figure 6a**), *Halomonas*, *PLTA13_ge*, *RB41*, *Elin6055*, *Gemmatimonadaceae_unclassified*, *Xanthobacteraceae_unclassified*, *Massilia*, *MND1*, *AKAU4049_ge*, *UTCFX1*, *Longimicrobiaceae_ge*, and *Gemmatimonadaceae_uncultured* were correlated with metabolites. *Halomonas* was significantly correlated with 21 metabolites, of which 12 were positively correlated and 9 were negatively correlated. *PLTA13_ge* was significantly correlated with 25 metabolites, of which 24 were positively correlated and only 1 metabolite was significantly negatively correlated. *RB41* was significantly correlated with 13 metabolites, of which 7 were positively correlated and 6 were negatively correlated. *Elin6055* was significantly correlated with 10 metabolites, of which 5 were positively correlated and 5 were negatively correlated. *Gemmatimonadaceae_unclassified* was significantly correlated with 17 metabolites, of which 12 were positively correlated and 5 were negatively correlated. *Xanthobacteraceae_unclassified* was significantly correlated with 20 metabolites, of which 15 were positively correlated and 5 were negatively correlated. *Massilia* was significantly correlated with 19 metabolites, of which 16 were positively correlated and 3 were negatively correlated. *MND1* was significantly correlated with 14 metabolites, of which 8 were positively correlated and 6 were negatively correlated. *AKAU4049_ge* was significantly correlated with 16 metabolites, of which 9 were positively correlated and 7 were negatively correlated. *UTCFX1* was significantly correlated with 4 metabolites, all of which were positively correlated. *Longimicrobiaceae_ge* was significantly correlated with 8 metabolites, all of which were positively correlated. *Gemmatimonadaceae_uncultured* was significantly negatively correlated with the 2 metabolites.

To ensure strong interaction, we constructed a co-expression network with a correlation coefficient greater than 0.95 or less than −0.95. There are interactions between microorganisms and plants. In medicinal plants, microorganisms in the soil have a remarkable relationship with the metabolites of medicinal plants. Our study found that *Acidobacteria*, *Proteobacteria*, *Gemmatimonadetes*, and *Chloroflexi* were correlated with metabolites at the phylum level (**Figure 6b**). A total of 13 metabolites were significantly correlated with *Acidobacteria*, of which 7 were negatively correlated (i.e., syringic acid, uridine diphosphate e-N-acetylglucosamine, 1-methylhistidine, carbofuran, dehydroepiandrosterone, glyceric acid, and N, N-dimethyl histidine) and 6 were positively correlated with Sophoradiol (e.g., 4,5-dihydroorotic acid, 2-acetamidoethylphosphonate, 5-L-glutamyl-L-glutar, and D-iditol). Proteobacteria was significantly correlated with five metabolites, all of which were negatively correlated, namely, L-arginine, D-fructose 1-phosphate, taxifolin, D-xylitol, and polygodial. Chloroflexi showed a significant correlation with four metabolites, all of which were positively correlated, namely, (2S,5S)-trans-carboxymethylp6Rliae, polygodial, D-xylitol, and L-4-hydroxyphenylglycine. Only L-formylkynurenine had a significant correlation with *Gemmatimonadetes*, and this correlation was negative.

### 3.4 Active ingredient analysis

**Figure 7:**
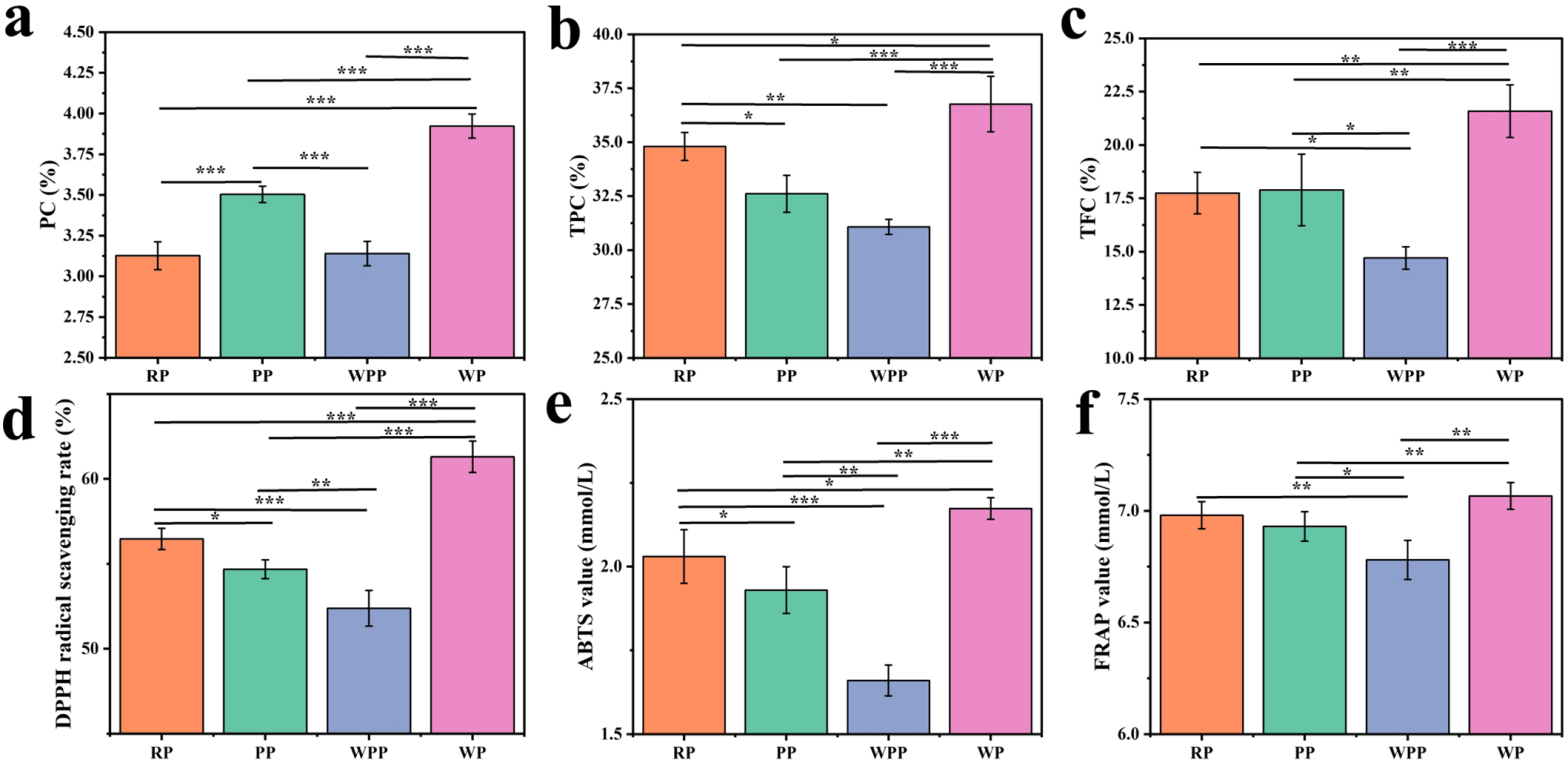
PC, TPC, and TFC and antioxidant activity. **(a)** PC. **(b)** TPC. **(c)** TFC. **(d)** DPPH free radical scavenging capacity. **(e)** ABTS free radical scavenging ability. **(f)** Total antioxidant capacity. *P<0.05, **P<0.01, ***P<0.001.

PC is the main component of *P. lactiflora*. According to the Chinese Pharmacopoeia 2020, PC in *P. lactiflora* should not be less than 1.8%. In **Figure 7a**, the PC in the four varieties was higher than that in the pharmacopeia. Among them, the PC in the WP group was the highest (3.92%), whereas the PC in the RP group was the lowest (3.13%). In general, WP > PP > WPP > RP. Except for the RP and WPP groups, the differences among the other groups were significant.

The rich TPC in peony gives it a variety of pharmacological activities and health benefits, supporting its wide application in traditional and modern medicine. **Figure 7b** shows that the TPC of each group differed; the highest TPC of 36.52% was found in WP, and the lowest TPC of 31.33% was found in WPP. In general, WP > RP > PP > WPP. Except for the WPP and PP groups, the difference between other groups was significant. Flavonoids are the active components of Chinese medicine in peony, which not only supports its wide application in traditional medicine but also provide a theoretical basis for its development as a modern drug and health product. As shown in **Figure 7c**, the difference in TFC between the RP and PP groups was small. WP had the highest TFC of 21.58%, and WPR had the lowest TFC of 14.70%. In general, WP > PP > RP > WPP. Except for the RP and PP groups, the difference among other groups was significant.

### 3.5 Antioxidant activity analysis

**Figures 7d–f** show that the four kinds of peonies exhibited varying antioxidant capacities. **Figure 7d** shows that the DPPH radical scavenging rate was 62.15%. RP, PP, and WPP were not significantly different from the WP group, with values of 55.95%, 54.63%, and 52.67%, respectively, but we found significant differences among the four groups. **Figure 7e** shows the antioxidant capacity of different herbaceous peonies on ABTS. The strongest antioxidant capacity was observed in the WP group (2.17 mmol/L), whereas the lowest content was found in the WPP group (1.66 mmol/L). Overall, WP > PP > RP > WPP, and there were significant differences among the four groups. **Figure 7f** shows the antioxidant capacity of different herbaceous peonies on FRAP. The total antioxidant capacity of the WR group was the strongest at 7.06 mmol/L, and the total antioxidant capacity of the WPR group was the weakest at 6.78 mmol/L. We found no significant difference between the RR and PR groups, which had values of 6.98 and 6.93 mmol/L, respectively. In addition to RR and PR, the differences in the RR and WR groups were not significant, but the other groups exhibited significant differences.

## 4 Discussion

Inter-root microorganisms are non-negligible factors affecting the growth of medicinal plants. On the one hand, inter-root microorganisms can promote plant growth; for example, inoculation with plant growth-promoting rhizobacteria improves the photosynthesis performance of seagrass *Thalassia hemprichii* (Zhou et al., 2024). On the other hand, harmful inter-root microorganisms can have negative effects on plants; for example, plant tumors can reduce crop yield and weaken disease resistance (Ullrich et al., 2019). Therefore, inter-root microorganisms play an important role in plant growth and development. In this study, we evaluated the differences in inter-root microbial species of different peony cultivars via the 16S rRNA technique. The results showed significant differences in the alpha diversity and beta diversity of the inter-root microbial communities of different peonies, and the distribution patterns of the taxonomic, functional, and phenotypic compositions of the inter-root microbiomes varied depending on the varieties; this result was similar to the study of different varieties of blueberries (Zhang et al., 2021b). Our results also revealed that Proteobacteria was the most abundant at the phylum level and *Halomonas* was the most abundant at the genus level; these findings were consistent with other studies on peonies (Yang et al., 2023a). *Actinomycetes* are saprophytic bacteria that can increase the disease resistance of plants (Khan et al., 2023; Oloumi et al., 2023).

Medicinal plant metabolites are the key to plant quality. Our study revealed a total of 207 differential metabolites in different species of *P. lactiflora*, with the phenolic compound species exhibiting the greatest abundance. This result was in agreement with previous studies (Yang et al., 2023b), which reported that different species of peonies have high numbers of phenolic and flavonoid metabolite species (Li et al., 2024). The interaction between plant roots and plant inter-root microorganisms is crucial for plant growth and quality. Co-occurrence network analysis showed a strong correlation between differentially abundant microorganisms and metabolites. Thus, metabolites can modify microbial species to adapt to the environment. Our study revealed that the species of metabolites were strongly correlated with *Acidobacteria* and *Halomonas*, which was similar to the findings of *Panax quinquefolium* (Shi et al., 2021). Total phenol, flavonoid, and procyanidin levels and total antioxidant activity of different Korean pine (*Pinus koraiensis*) varieties have been studied [39], demonstrating differences. The contents of active ingredients and free radical scavenging capacity of different onion varieties also differed (Zhang et al., 2019), which was consistent with our findings.

## 5 Conclusions

In summary, this study integrated physiological, microbiome, and metabolomics techniques and multivariate data methods to analyze the differences among four different flowering peony varieties. Results showed that *Halomonas* was the highest at the genus level, and Proteobacteria was the highest at the phylum level. Among the metabolite differences, there were 207 differential metabolites. The phenolic compounds exhibited the most significant differences, and we observed a relationship between microbial communities and metabolites. The PC, TPC, and TFC were the highest in WP, indicating that it had the strongest antioxidant capacity among the groups.

## Data Availability

The datasets presented in this study can be found in online repositories. The names of the repository/repositories and accession number(s) can be found in the article/**Supplementary Material**.

## Supplementary material

Supplementary Figure 1 KEGG Venn diagram.

Supplementary Table 1 Illumina sequencing result.

Supplementary Table 2 Analyzed the composition of rhizosphere microorganisms at the phylum levels.

Supplementary Table 3 Analyzed the composition of rhizosphere microorganisms at the genus levels.

Supplementary Table 4 LEfSe analysis.

Supplementary Table 5 All metabolites.

Supplementary Table 6 Differential metabolites.
