# Supplementary figures and images for: Integrated physiological, microbial, and metabolomics analyses revealed the differences in different varieties of Paeonia lactiflora Pall
Source: Front Plant Sci. 2025 May 23;16:1577695. doi: 10.3389/fpls.2025.1577695 (PMC12141275; doi:10.3389/fpls.2025.1577695)

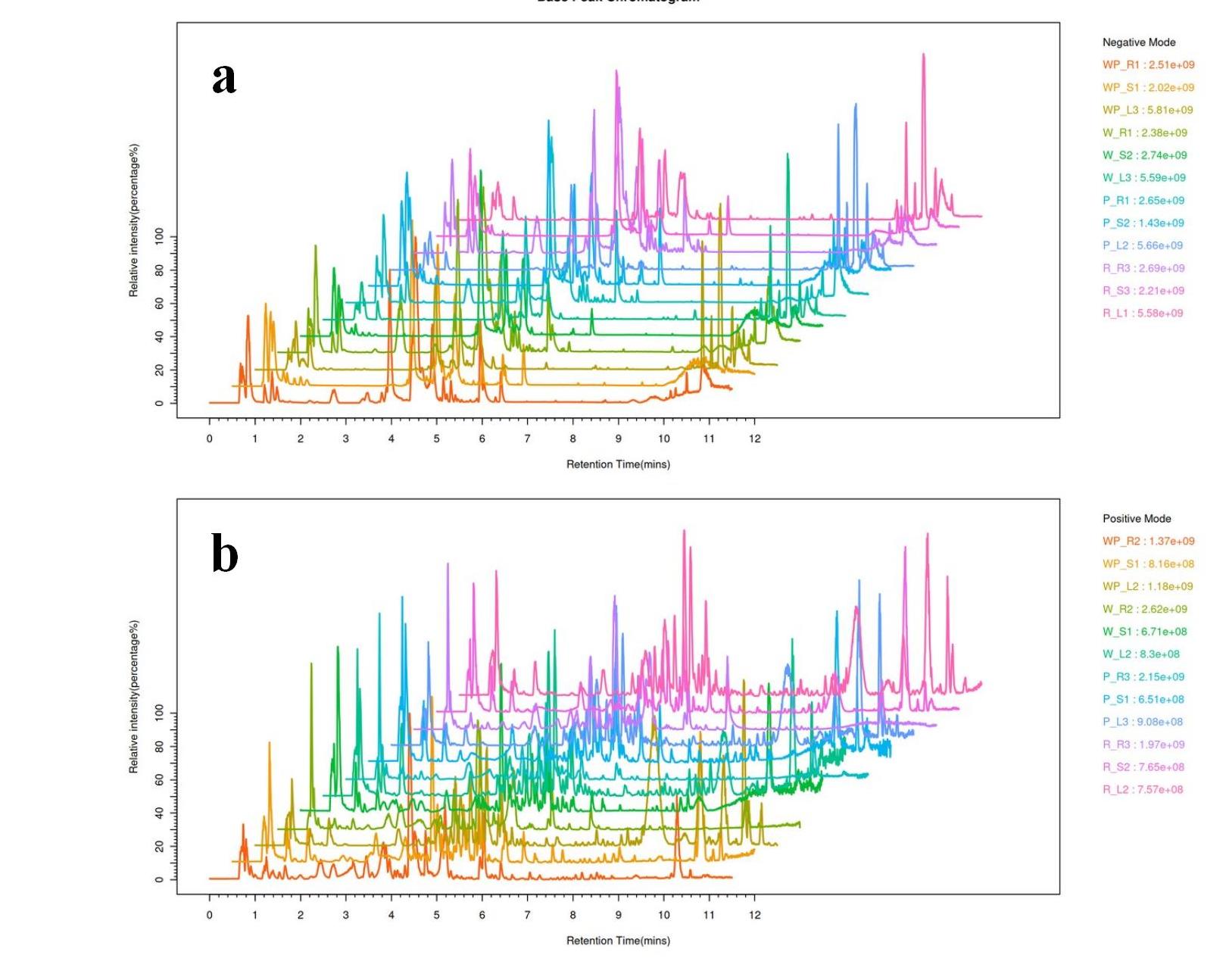

Supplement: Supplementary Figure 1 — KEGG Venn diagram. [file Image1.jpeg]

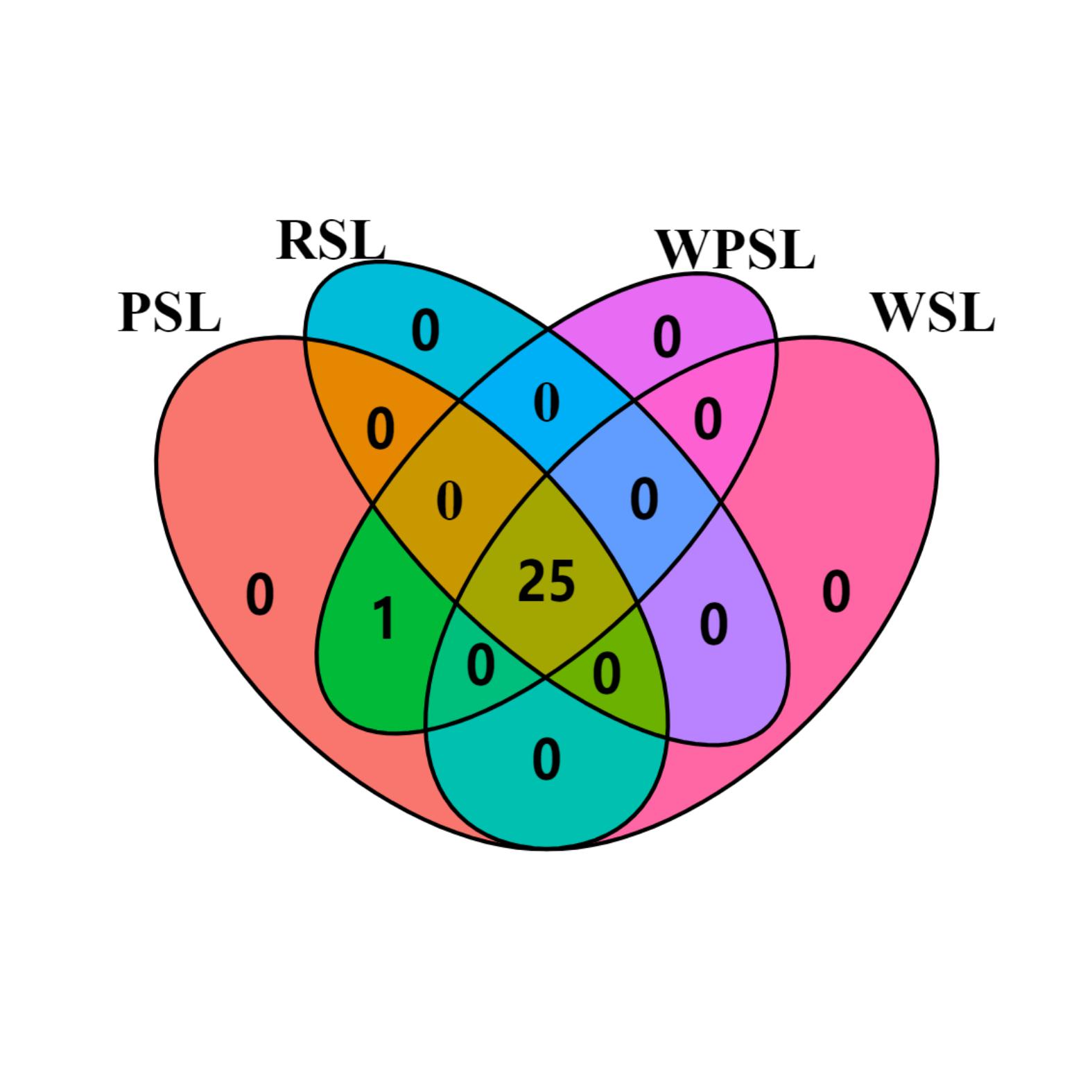

Supplement: Supplementary file 2 [file Image2.jpeg]

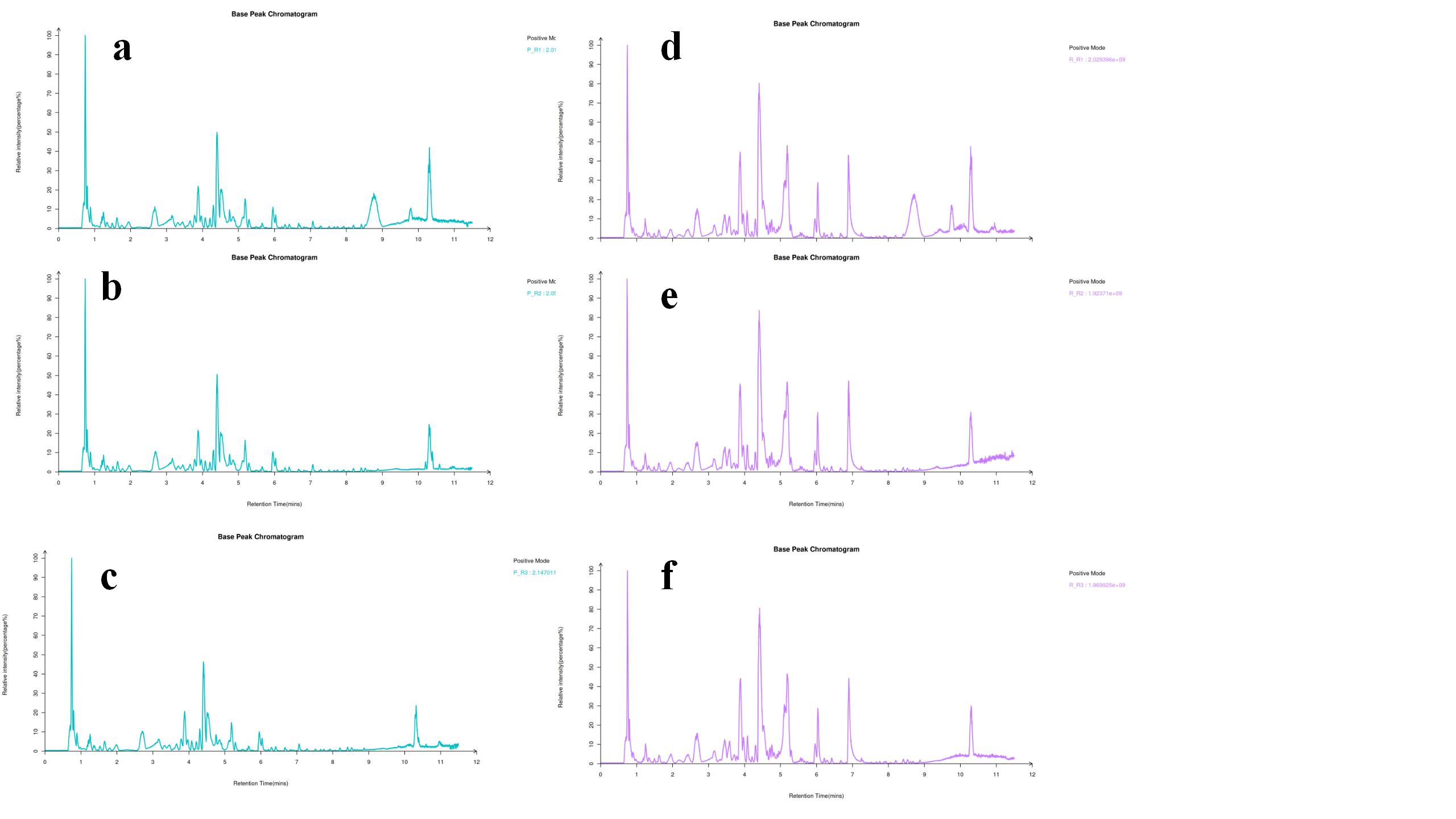

Supplement: Supplementary file 3 [file Image3.jpeg]

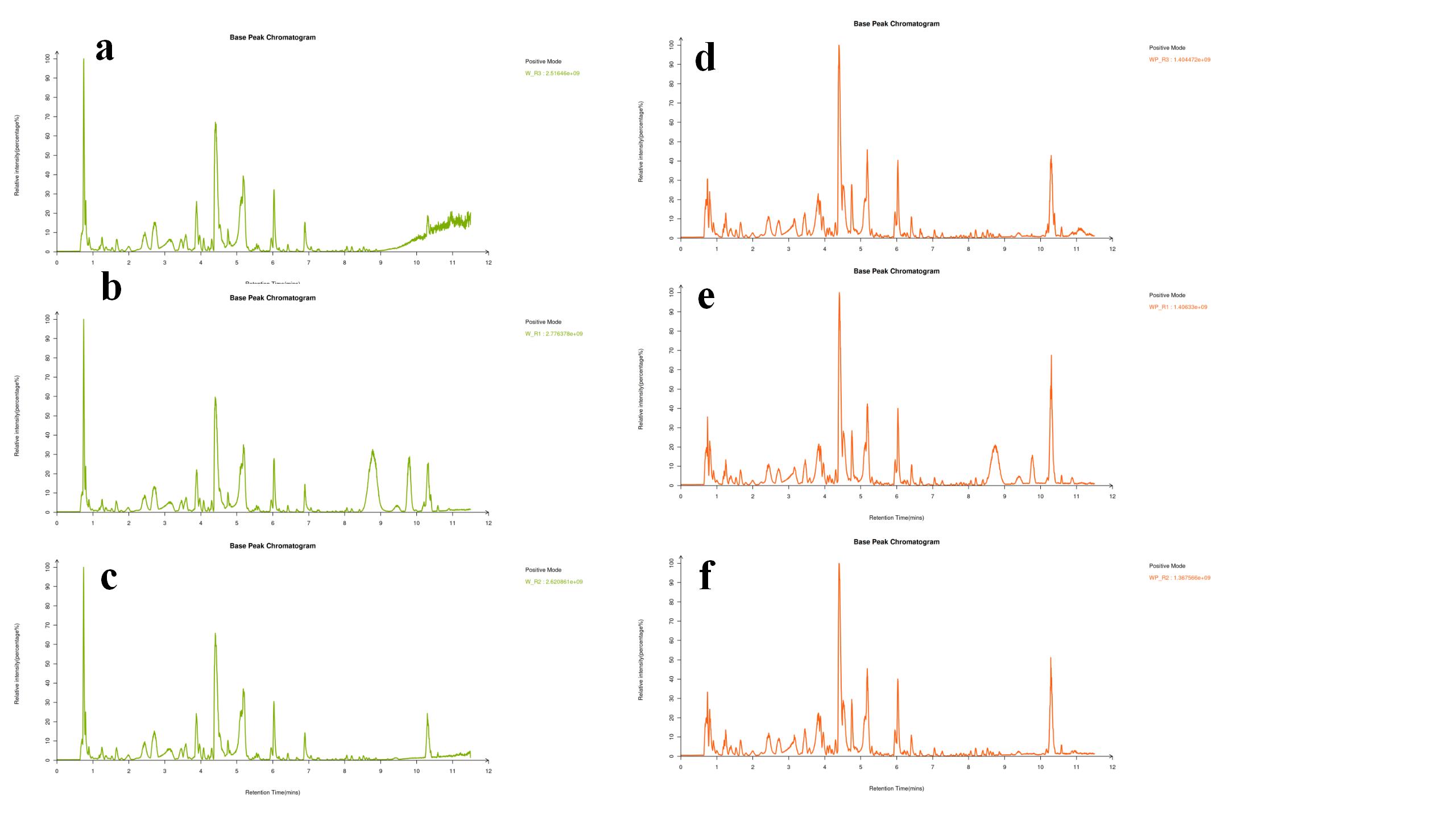

Supplement: Supplementary file 4 [file Image4.jpeg]

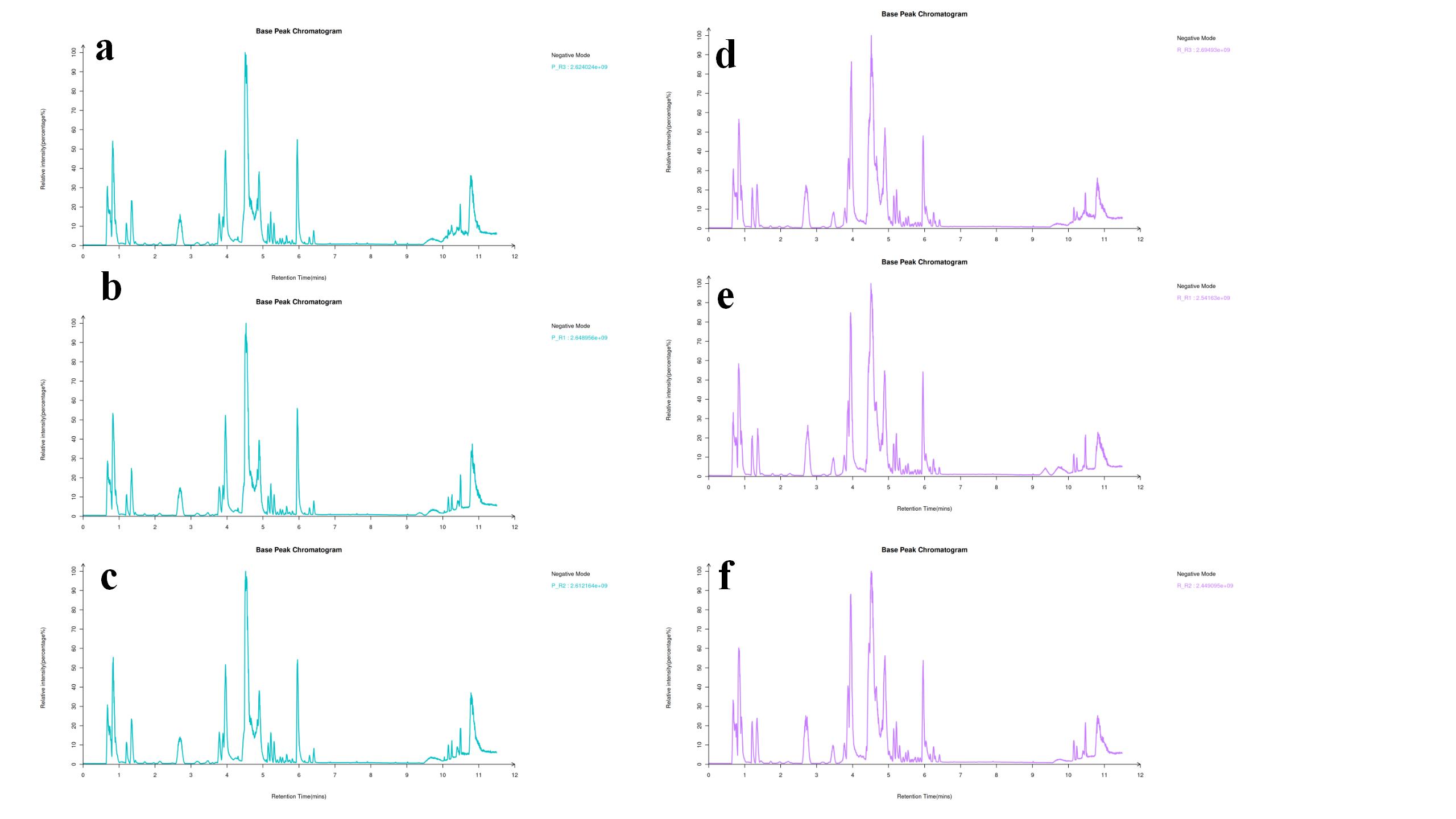

Supplement: Supplementary file 5 [file Image5.jpeg]

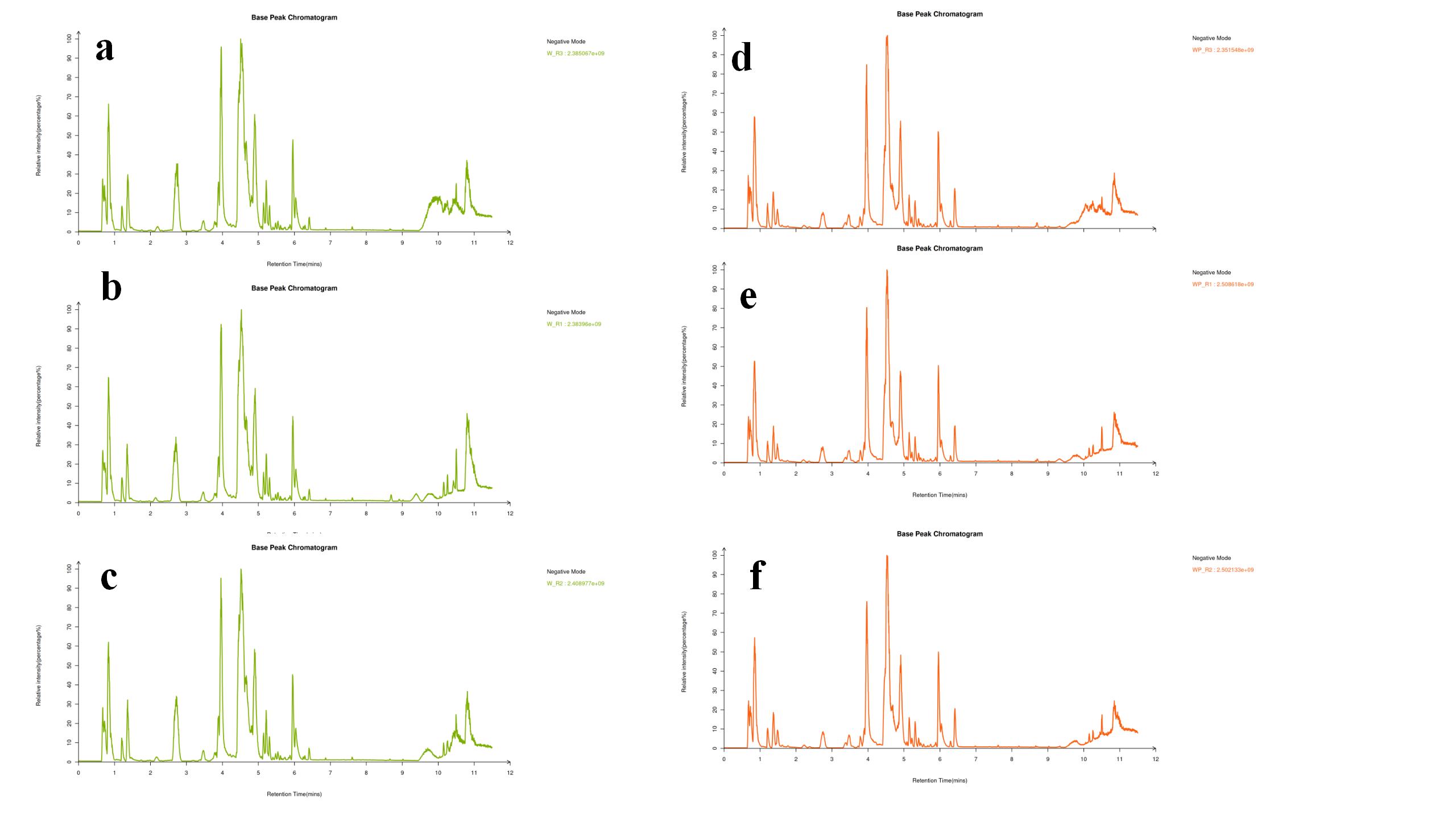

Supplement: Supplementary file 6 [file Image6.jpeg]
